# Single-Cell Transcriptomic Profiling Reveals Immunometabolic Reprogramming and Cell-Cell Communication in the Tumor Microenvironment of Human Hepatocellular Carcinoma

**DOI:** 10.3390/ijms27125397

**Published:** 2026-06-15

**Authors:** Miguel Ángel Díaz-Campos, Enrique Hernández-Lemus

**Affiliations:** 1 Graduate Program in Biochemical Sciences, Universidad Nacional Autónoma de México, Mexico City 04510, Mexico; g5diazcamposmiguel@gmail.com; 2 Computational Genomics Division, Instituto Nacional de Medicina Genómica, Mexico City 14610, Mexico

**Keywords:** hepatocellular carcinoma, single-cell transcriptomics, tumor microenvironment, immune reprogramming, cell–cell communication, immunometabolic reprogramming

## Abstract

Hepatocellular carcinoma (HCC) is sustained by coordinated interactions among malignant hepatocytes, immune cells, and stromal populations that collectively drive tumor growth, immune evasion, and vascular remodeling. Using integrative single-cell transcriptomics on 93,032 cells from tumor and healthy human liver, we characterized cell-type-specific transcriptional programs underlying immunometabolic reprogramming and reconstructed the intercellular communication circuits that maintain the tumor microenvironment. Malignant hepatocytes displayed upregulation of genes encoding both glycolytic and oxidative phosphorylation (OXPHOS) metabolic enzymes, consistent with metabolic plasticity, while concurrently suppressing genes involved in antigen presentation—a transcriptional pattern indicative of coordinated metabolic and immune-evasive reprogramming. Tumor-associated macrophages acquired *TREM2*-enriched, lipid-handling phenotypes consistent with immunosuppressive polarization, and tumor endothelial cells upregulated angiocrine and extracellular matrix programs while silencing innate immune outputs. Ligand–receptor inference revealed a qualitative rewiring of intercellular communication: the antigen-presentation-centered network of the healthy liver was replaced by a tumor-driven architecture dominated by pro-angiogenic, ECM–integrin, inflammatory chemokine, and lipid-associated signaling circuits, with malignant hepatocytes, TAMs, and TECs collectively assuming the dominant signaling burden. These findings establish that HCC progression is an emergent property of a stabilized multicellular network, rather than the autonomous behavior of malignant cells, and define cooperative immunometabolic modules that constitute tractable targets for combinatorial therapeutic intervention.

## 1. Introduction

Hepatocellular carcinoma (HCC) is the most prevalent primary liver malignancy and a leading cause of cancer-related mortality worldwide [1]. It arises predominantly in the context of chronic liver disease, with hepatitis B and C virus infections, alcoholic liver disease, and non-alcoholic steatohepatitis as the principal etiological drivers [2,3]. Late diagnosis, high recurrence rates after resection or ablation, and limited efficacy of systemic therapies in advanced disease contribute to persistently poor outcomes [4,5]. Despite improvements in diagnostic imaging, the cellular architecture of the HCC tumor microenvironment (TME) and its contribution to disease progression remain incompletely characterized [2,5].

The liver comprises a structured mixture of parenchymal and non-parenchymal cells, including hepatocytes, sinusoidal endothelial cells, Kupffer cells, hepatic stellate cells, and diverse immune subsets [6]. Chronic inflammation and fibrotic remodeling disrupt this tightly regulated environment, shifting cellular composition, immune activity, and metabolic function toward a permissive oncogenic context [7,8]. Tumor progression is not driven solely by malignant hepatocytes but is profoundly shaped by surrounding stromal and immune populations that can suppress or facilitate growth [6,7].

The HCC TME is defined by three coordinated processes: immunosuppression, neoangiogenesis, and metabolic adaptation. Tumor-associated macrophages (TAMs) blunt cytotoxic immunity by secreting anti-inflammatory mediators and inhibiting lymphocyte activity. Tumor endothelial cells (TECs) drive vascular remodeling while attenuating immune surveillance at the sinusoidal interface. Malignant hepatocytes reprogram their metabolic state, enhancing glycolysis, increasing mitochondrial respiration, and acquiring resistance to oxidative stress, to sustain proliferation under nutrient-deprived conditions [9]. Whether these processes are mechanistically coupled through cell–cell communication or operate in parallel remains unresolved.

Single-cell RNA sequencing (scRNA-seq) has begun to reshape our understanding of HCC biology by enabling cell-type-resolved analysis of transcriptional states within the tumor microenvironment. Xie et al. reconstructed a global single-cell landscape of HCC from 27 tumor and 4 adjacent non-tumor tissues, identifying a novel HP-expressing hepatocellular cluster and an *AIF1*^+^ neutrophil subpopulation with extensive intercellular communication with tumor cells, endothelial cells, and cancer-associated fibroblasts, and demonstrating pervasive upregulation of ErbB and GnRH signaling pathways across TME compartments [10]. Ye et al. integrated single-cell, spatial, and bulk multi-omics from six HCC cases, revealing that intratumoral heterogeneity is primarily driven by malignant hepatocyte diversity, and that HCC cells act as the core orchestrators of pro-tumorigenic TAM polarization, while endothelial cells execute spatially distinct functions across the tumor ecosystem [11]. Guo et al. analyzed 52 scRNA-seq datasets and identified three transcriptionally distinct malignant subtypes, an ARG1^+^ metabolic subtype, a TOP2A^+^ proliferative phenotype, and an S100A6^+^ pro-metastatic subtype, tracing a trajectory in which both the metabolic and EMT subtypes originate from the proliferative state. The EMT subtype, driven by exclusive SMAD3/TGF-β activation, was linked to poor prognosis through a positive feedback loop with fibroblasts mediated by SPP1–CD44 and CCN2–TGFBR1 signaling pairs. Dai et al. combined scRNA-seq with weighted gene co-expression network analysis and bulk transcriptomics to show that lipid metabolism reprogramming, centered on PTGES3, modulates the HCC immune microenvironment by potentiating FN1/CD44 and MDK/NCL immunosuppressive signaling cascades, associated with diminished immunotherapy responsiveness and adverse overall survival [12].

Collectively, these studies have illuminated key aspects of HCC cellular heterogeneity, metabolic rewiring, and selective communication circuits. Nevertheless, the simultaneous characterization of immunometabolic reprogramming, cell-type-specific transcriptional programs, and intercellular communication networks, benchmarked against healthy liver tissue, remains limited. Most prior analyses have addressed these axes independently, employed small cohorts, or lacked a healthy-tissue reference that would permit rigorous identification of disease-specific transcriptional changes. Moreover, the cooperative mechanisms through which malignant hepatocytes, TAMs, and tumor endothelial cells co-establish a stable immunosuppressive ecosystem through coordinated ligand–receptor signaling have not been systematically resolved. Addressing these gaps requires a multi-sample, multi-condition design with consensus-based communication inference applied simultaneously across compartments.

**Figure 1:**
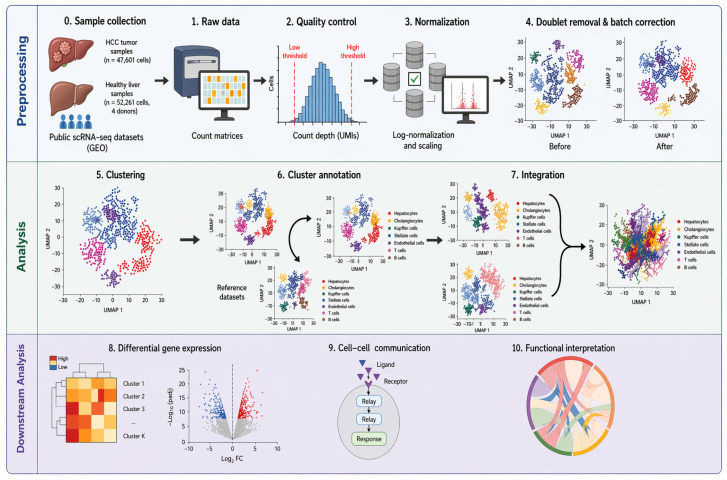
Schematic overview of the integrated single-cell transcriptomic pipeline for HCC characterization. The workflow encompasses data integration of five public scRNA-seq datasets, quality control (QC) filtering based on mitochondrial content and gene detection thresholds, doublet removal via Solo, consensus cell-type annotation combining CellTypist and scANVI classifiers, differential gene expression (DGE) analysis, Gene Set Enrichment Analysis (GSEA) using reference-based gene signatures, cancer stem cell (CSC) scoring and stratification, and multi-method consensus intercellular communication inference via LIANA (integrating CellChat, CellPhoneDB, SingleCellSignalR, and NATMI). Final outputs include integrated single-cell atlases, validated cell-type assignments, gene expression signatures, and ligand–receptor interaction networks that collectively define the immunometabolic landscape of the HCC tumor microenvironment.

Here, we integrate scRNA-seq data from tumor and healthy human liver cells to simultaneously characterize transcriptional states, metabolic programs, and intercellular communication networks in the HCC TME. Applying probabilistic cell annotation, pseudo-bulk differential expression, pathway enrichment, and consensus-based ligand–receptor inference, we delineate the cooperative immunometabolic modules that sustain malignant progression and identify the communication architecture that distinguishes the tumor ecosystem from homeostatic liver tissue (Figure 1).

## 2. Results

### 2.1. Cellular Remodeling of the Tumor Microenvironment

**Figure 2:**
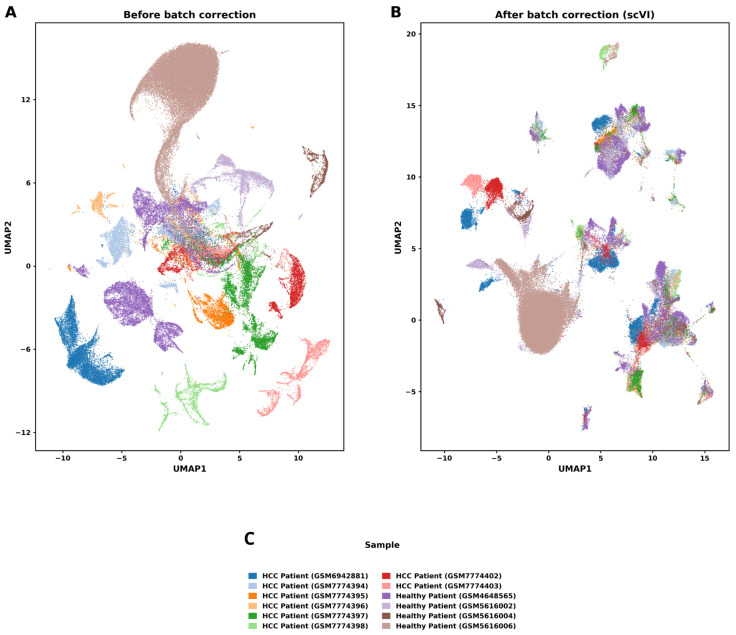
**Healthy and tumor samples before and after data integration.** (**A**) UMAP projection of single-cell transcriptomes labeled by patient of origin (4 healthy donors and 8 HCC patients) before batch correction. (**B**) UMAP after integration using scVI, showing well-mixed populations across donors after batch effects corrected. Integration enables joint analysis of cells across conditions. (**C**) Sample legend and donor metadata, indicating the healthy donors and HCC patients from public data included in the study.

Cross-sample data integration using scVI successfully aligned transcriptomes across all 12 samples (4 healthy donors and 8 HCC patients), with batch effects evaluated using PCA and UMAP before and after correction (Figure 2). The integration preserved biological variability while removing technical batch artifacts, enabling robust comparison of cellular states across conditions.

**Figure 3:**
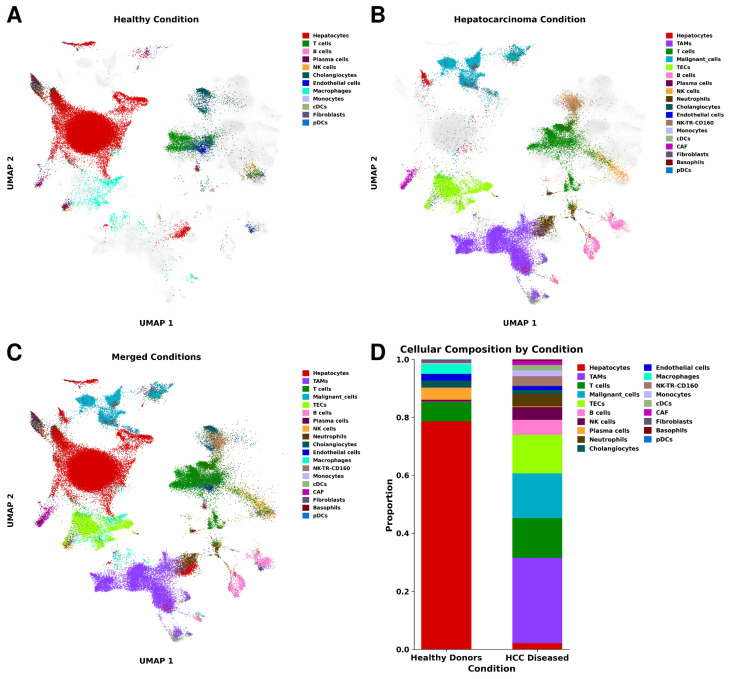
**Cell-type composition across healthy and hepatocellular carcinoma (HCC) liver samples.** Profiles from healthy and tumor liver tissues were integrated and annotated to characterize the cellular landscape of both conditions. Cell identities were assigned using a reference-based annotation strategy combining CellTypist and scANVI, allowing consistent identification of immune, stromal, and epithelial populations across datasets. (**A**) UMAP projection of cells derived from healthy liver samples after cell-type annotation, revealing major hepatic and immune populations including hepatocytes, cholangiocytes, endothelial cells, fibroblasts, macrophages, neutrophils, B cells, T cells, natural killer (NK) cells, and dendritic cells. (**B**) UMAP projection of cells derived from hepatocellular carcinoma samples. In addition to immune and stromal populations, malignant epithelial cells and tumor-associated populations such as tumor-associated macrophages (TAMs), cancer-associated fibroblasts (CAFs), and tumor endothelial cells (TECs) are observed, reflecting the complexity of the tumor microenvironment. (**C**) Integrated UMAP embedding combining healthy and tumor-derived cells following batch correction and latent space integration, enabling direct comparison of cellular states across physiological and tumor conditions, and highlighting shared and condition-specific cell populations. (**D**) Comparison of cell-type proportions between healthy and HCC conditions. Tumor samples exhibit an increased abundance of malignant epithelial cells and tumor-associated stromal populations, together with shifts in immune cell composition indicative of remodeling of the hepatic tumor microenvironment.

Healthy liver samples displayed the expected parenchymal-dominant composition, with hepatocytes comprising 80.6% of the healthy fraction—a proportion consistent with the architecture of a histologically normal liver—supported by macrophages, endothelial cells, and lymphoid subsets (Figure 3) [13,14]. HCC samples exhibited a marked shift in cellular representation: malignant epithelial cells expanded substantially (29.7% of tumor-derived cells), accompanied by proportional increases in tumor-associated macrophages (TAMs; 26.7%) and tumor endothelial cells (TECs; 20.0%), while hepatocytes were reduced to 4.1% (Figure S1). This compositional remodeling is consistent with the progressive displacement of homeostatic hepatic architecture by tumor-supportive stromal and immune compartments [14,15,16].

### 2.2. Immunometabolic Reprogramming of Malignant Hepatocytes

**Figure 4:**
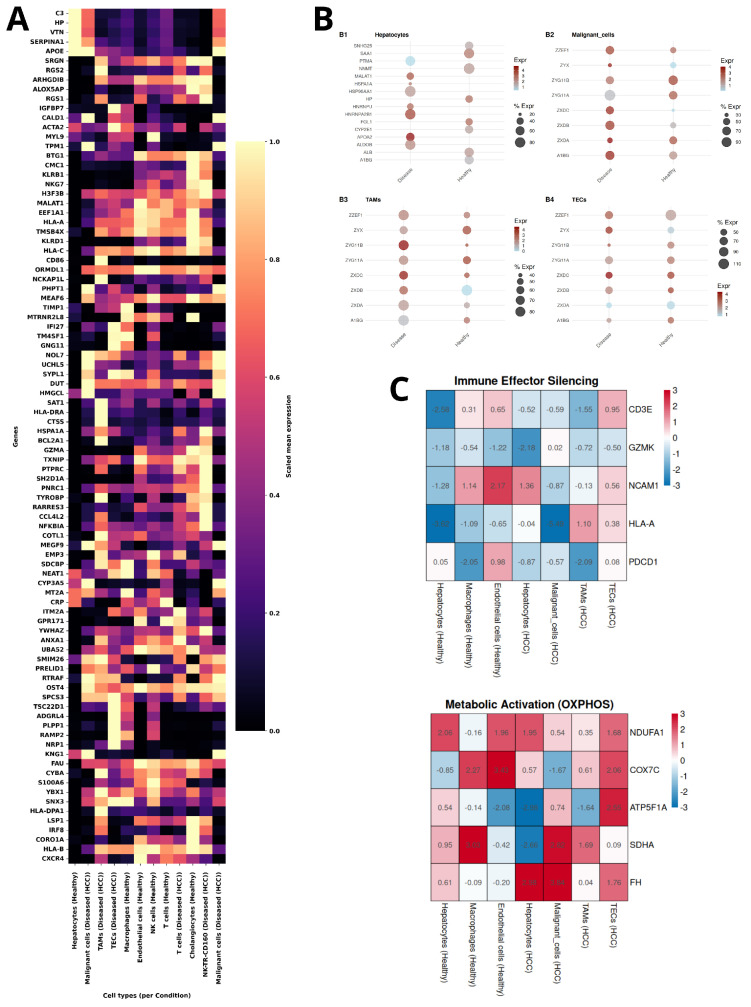
**Transcriptional signatures of cell-type-specific reprogramming in hepatocellular carcinoma (HCC).** (**A**) Scaled mean expression of canonical marker genes across the major annotated cell populations identified in healthy donors and HCC patients. Genes are organized by cell type and highlight lineage-specific transcriptional programs across parenchymal, immune, endothelial, and stromal compartments, including hepatocyte markers (*ALB*, *APOA1*), macrophage/TAM signatures (*MARCO*, *TREM2*, *GPNMB*), and endothelial markers (*PECAM1*, *CDH5*, *KDR*). (**B**) (**B1**) Malignant hepatocytes upregulate oncogenic and stress-response transcripts (*DUXAP8*, *MALAT1*, *HELLPAR*) while downregulating canonical parenchymal markers (*ALB*, *ALDOB*, *APOA2*, *CYP2E1*), reflecting the dismantling of hepatocellular metabolic identity during malignant transformation. (**B2**) Malignant cells are dominated by cell-cycle and chromosomal instability signatures with concurrent suppression of immune effector and cell-adhesion programs, defining an oncogenic state that couples unrestrained proliferation with immune evasion. (**B3**) TAMs upregulate lipid-sensing, complement-associated (*C1QB*, *C1QC*), and immunomodulatory transcripts (*TREM2*, *PLA2G7*) while losing homeostatic surveillance programs (*MARCO*), consistent with pro-tumorigenic polarization toward an immunosuppressive niche. (**B4**) TECs upregulate angiogenic and matrix-remodeling factors (*KDR*, *SPARC*, *COL4A1*) alongside downregulation of lymphocyte-interaction mediators (*GZMK*, *CD3E*), reflecting an angiocrine switch toward vascular remodeling and immune silencing. (**C**) Metabolic–immune axis heatmaps spanning healthy and HCC-diseased cell populations. (**Top**) Immune effector genes (*CD3E*, *GZMK*, *NCAM1*, *HLA-A*, *PDCD1*) showing suppressed expression in HCC-diseased states across all populations, defining the immunometabolic switch that characterizes tumor-associated cell reprogramming. (**Bottom**) Oxidative phosphorylation genes (*NDUFA1*, *COX7C*, *ATP5F1A*, *SDHA*, *FH*) showing elevated expression in HCC conditions: hepatocytes (HCC), malignant cells (HCC), TAMs (HCC), and TECs (HCC), compared to their healthy counterparts: hepatocytes (healthy), macrophages (healthy), and endothelial cells (healthy).

Pseudo-bulk differential expression analysis identified extensive transcriptional divergence between malignant and non-malignant hepatocytes (Figure 4A and Figure S2) [17,18]. Among the most strongly upregulated transcripts were long non-coding RNAs including *DUXAP8*, *HELLPAR*, and *LINC00470*, together with *PRRT4* and *GALNT9*. These transcripts have been implicated in oncogenic transcriptional programs and cell proliferation in hepatic malignancy [19,20]. Concurrent downregulation of metallothionein family members (*MT1G*, *MT1F*, *MT1M*, *MT1E*, *MT1A*) and the iron-regulatory peptide *HAMP* indicates disruption of metal homeostasis and cytoprotective responses—hallmarks of hepatocyte dedifferentiation [21]. Together, these transcriptional changes delineate a coordinated dismantling of hepatocyte-specific metabolic identity, encompassing metal detoxification, iron homeostasis, and redox-protective programs that are hallmarks of functional parenchymal differentiation.

Functional enrichment of downregulated genes revealed selective repression of ribosomal biogenesis and cytoplasmic translation programs, encompassing terms such as cytosolic ribosomal subunit, structural constituent of ribosome, and cytoplasmic translation. This transcriptional pattern indicates a fundamental restructuring of the biosynthetic apparatus in malignant hepatocytes, consistent with the metabolic reallocation from protein quality control toward anabolic and proliferative outputs [21,22]. Downregulation of cell substrate junctioncomponents further supports disruption of hepatocyte-matrix anchoring, a prerequisite for invasive behavior.

Upregulated gene sets were enriched for chromatin-associated and nuclear organizational programs, including chromosomal region, nuclear body, protein–DNA complex, nuclear chromosome, DNA metabolic process, and ATP hydrolysis activity. This pattern indicates broad nuclear reorganization and chromatin restructuring in malignant hepatocytes, consistent with the genomic instability and epigenetic reprogramming that characterize hepatocellular transformation [19,20]. Together, the repression of ribosomal biogenesis programs combined with induction of chromosomal and DNA metabolic gene sets reflects the transcriptional logic of malignant transformation: dismantling hepatocyte biosynthetic identity while engaging chromatin-level proliferative programs [19,20]. Concurrent downregulation of MHC class I loading components further links metabolic reprogramming to diminished antigen presentation capacity, suggesting that metabolic and immune-evasive transcriptional patterns are coordinately downregulated in malignant hepatocytes [23].

### 2.3. Proliferative and Immune-Evasive Programs in Malignant Cells

Pseudo-bulk analysis of malignant hepatocellular cells relative to healthy hepatocytes identified a distinct transcriptional program reflecting oncogenic cell-cycle engagement and immune avoidance (Figure S3). Upregulated gene sets were strongly enriched for mitotic and chromosomal programs, including chromosome organization, chromosome segregation, mitotic sister chromatid segregation, cell cycle process, organelle fission, and cell cycle terms. This coordinated upregulation of cell-division machinery is consistent with the unrestrained proliferative capacity and chromosomal instability characteristic of advanced HCC [19,20].

Downregulated gene sets in malignant cells encompassed molecular transducer activity, cell adhesion, defense response, inflammatory response, cell activation, cytokine production, and regulation of immune system process terms. The enrichment of downregulated genes within immune effector and cell-adhesion programs indicates transcriptional silencing of immune recognition and intercellular communication pathways, facilitating immune escape and acquisition of migratory competence [17,23]. Together, the transcriptional profile of malignant cells, dominated by cell-cycle activation and concurrent downregulation of immune effector gene sets, defines an oncogenic state that couples proliferative drive with immune evasion—a configuration that fundamentally reorganizes the tumor microenvironment to favor tumor progression.

### 2.4. Cancer Stem Cell Subpopulations Within Malignant Hepatocellular Cells

To address the question of intratumoral heterogeneity within the malignant compartment, we performed a dedicated subpopulation analysis aimed at identifying cancer stem cell (CSC)-like states among the 6302 malignant cells detected in our dataset. CSCs have been increasingly recognized as drivers of HCC progression, drug resistance, and immune evasion [24,25], yet their transcriptional identity at single-cell resolution remains incompletely characterized.

**Figure 5:**
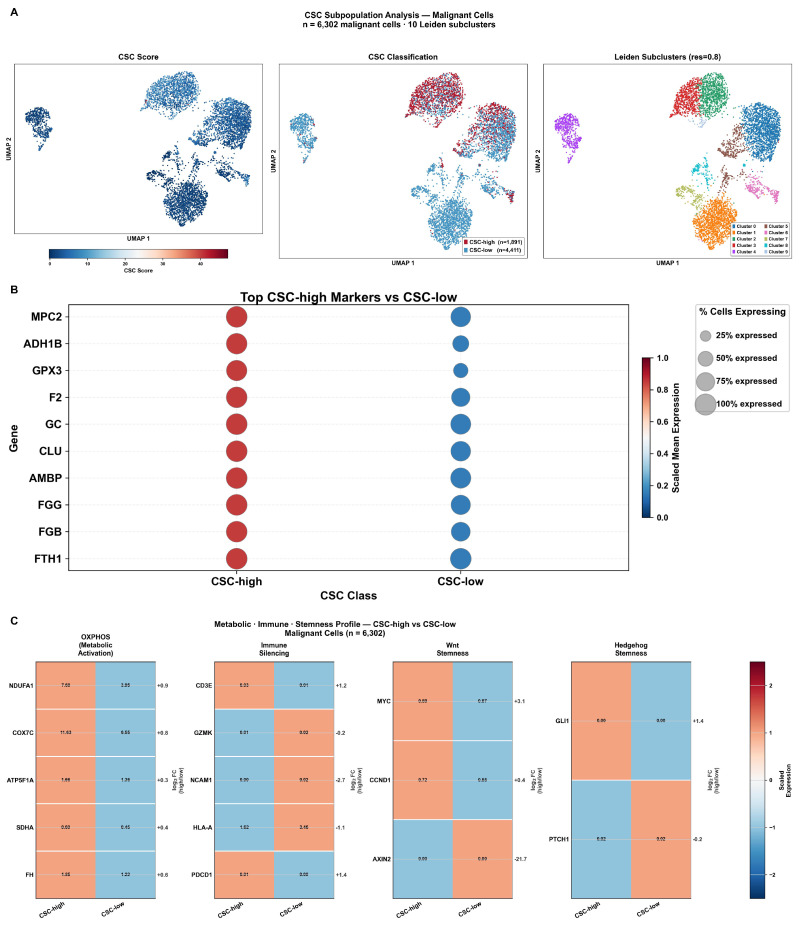
**Cancer stem cell (CSC) subpopulations within malignant hepatocytes exhibit distinct transcriptional and metabolic signatures.** (**A**) **Left**: UMAP projection of 6302 malignant hepatocytes colored according to their continuous CSC score. **Center**: Binary classification of malignant cells into CSC-high (*n* = 1891) and CSC-low (*n* = 4411) populations based on CSC scoring. **Right**: Leiden subclustering analysis (resolution = 0.8) identifying 10 transcriptionally distinct malignant cell subpopulations. The distribution of CSC-high cells across multiple subclusters indicates possible stemness-associated programs enriched in specific malignant cell states rather than defining a single homogeneous population. (**B**) Top differentially expressed genes between CSC-high and CSC-low populations. CSC-high cells are enriched for stem-related markers including *FTH1* (ferritin heavy chain), fibrinogen subunits (*FGB*, *FGG*), and secreted proteins (*AMBP*, *CLU*), while CSC-low cells express higher levels of differentiated hepatocyte and metabolic markers. (**C**) Metabolic–immune gene panel heatmap comparing mean expression across four functional categories: oxidative phosphorylation (OXPHOS), immune effector, Wnt pathway, and Hedgehog pathway genes. CSC-high cells exhibit reduced OXPHOS expression, suppressed immune effector genes, and elevated stemness pathway signaling compared to CSC-low cells, reflecting a distinct transcriptional state with reduced oxidative capacity and enhanced self-renewal potential.

We defined a composite CSC gene signature by integrating three complementary sources: (i) canonical surface markers validated in HCC (*CD44*, *EPCAM*, *PROM1*/CD133, *ALDH1A1*, *CD24*), (ii) the 17-gene CNV-based stemness signature identified by Ding et al. (2025) [25] from scRNA-seq analysis of HCC (*HAMP*, *GPC3*, *DNAJC6*, *NT5DC2*, *UBD*, *ATAD2*, *LAMC1*, *GABRE*, *LRRC1*, *MUC13*, *STK39*, *SDS*, *PPP1R1A*, *TRIM22*, *FGFR2*, *SPINK1*, *IGF2BP2*), and (iii) developmental genes identified through Monocle3 pseudotime trajectory analysis by Lin et al. (2024) [24] (*HSPB1*, *ADH4*, *FTH1*, *APCS*). Of the 35 candidate genes, 34 were detected in our dataset. Cell-level CSC scores were computed using sc.tl.score_genes, and cells scoring in the top 30% were classified as CSC-high (n=1891; 30%), while the remaining cells were designated CSC-low (n=4411; 70%) (Figure 5A). CSC-high cells are operationally defined as those malignant cells displaying a transcriptional state consistent with stemness: elevated co-expression of self-renewal, plasticity, and stress-resistance genes that collectively confer tumor-initiating capacity, therapy resistance, and metastatic potential. CSC-low cells, in contrast, represent the more differentiated bulk of the malignant compartment, characterized by active proliferation but reduced self-renewal capacity and greater sensitivity to cytotoxic stimuli.

Leiden subclustering of the malignant compartment at resolution 0.8 yielded 10 transcriptionally distinct subpopulations (Figure 5A, right). CSC-high cells were not uniformly distributed but were concentrated in specific Leiden subclusters, indicating that CSC-like states represent a discrete transcriptional identity rather than a continuous gradient. Differential expression analysis between CSC-high and CSC-low populations identified *FTH1* (Ferritin Heavy Chain 1) as the top CSC-high marker (Wilcoxon score =52.6, log_2_FC =154.6, $p_{\mathrm{adj}}<10^{-300}$), followed by fibrinogen subunits *FGB* and *FGG*, and secreted proteins *AMBP*, *CLU*, *GC*, and *F2* (Figure 5B). The prominence of *FTH1*, an iron storage protein whose overexpression promotes stemness maintenance through ferroptosis resistance, is consistent with emerging evidence linking iron metabolism to CSC survival in HCC [24].

Metabolic profiling revealed divergent patterns between CSC-high and CSC-low cells across four functional gene panels (Figure 5C). CSC-high cells showed reduced expression of OXPHOS genes (*NDUFA1*, *COX7C*, *ATP5F1A*, *SDHA*, *FH*) relative to CSC-low cells, a transcriptional pattern consistent with reduced oxidative phosphorylation capacity. While this signature has been associated with stemness and glycolytic flexibility in prior studies [24], we note that gene expression signatures represent transcriptional proxies that do not directly measure glucose consumption rates or mitochondrial ATP production. Immune effector genes (*CD3E*, *GZMK*, *NCAM1*, *HLA-A*, *PDCD1*) were more suppressed in CSC-high cells, supporting an immune-evasive phenotype. Wnt and Hedgehog stemness pathway genes (*MYC*, *CCND1*, *AXIN2*, *GLI1*, *PTCH1*) showed enrichment in CSC-high cells, corroborating the activation of canonical stem cell self-renewal circuits in this subpopulation.

### 2.5. Pro-Tumorigenic Polarization of Tumor-Associated Macrophages

TAMs exhibited a transcriptional program diverging substantially from macrophages in non-tumoral liver tissue (Figure 4A,B; Figure S4) [26]. Upregulated genes included complement components *C1QB* and *C1QC*, the immunomodulatory phospholipase *PLA2G7*, the interferon-stimulated gene *ISG15*, and the scavenger receptor-associated transcript *TREM2*, together with *MS4A7* and *FCGR2A* (Figure 4B). This expression profile delineates a macrophage state specialized for lipid sensing, complement-mediated opsonization, and immunoregulatory signaling [27,28]. Downregulated transcripts included *MARCO*—a pattern recognition receptor associated with homeostatic immune surveillance—and the matricellular genes *POSTN* and *CTGF*, indicating loss of tissue-maintenance programs and extracellular matrix homeostatic functions (Figure 4B).

Functional enrichment of TAM upregulated genes identified activation of mitochondrial metabolic programs, encompassing aerobic respiration, cellular respiration, energy derivation by oxidation of organic compounds, and oxidoreductase activity, indicating broad enhancement of oxidative phosphorylation capacity in tumor-associated macrophages (Figure 4C, bottom panel). Conversely, downregulated gene sets in TAMs were enriched for cytosolic ribosomal programs alongside vascular remodeling terms including tube development, blood vessel morphogenesis, and vasculature development [29].

Together, the *TREM2*-enriched, lipid-scavenging, complement-expressing TAM profile is consistent with an immunometabolically active macrophage state documented in HCC [26,27]. Induction of mitochondrial oxidative programs alongside enrichment of downregulated gene sets within translational and vascular remodeling categories indicates that TAMs in HCC undergo metabolic reprogramming toward oxidative phosphorylation, a configuration that supports sustained immunosuppressive effector function within the tumor-supportive niche [28,29].

### 2.6. Angiogenic and Immune-Silent Reprogramming of Tumor Endothelial Cells

TECs displayed a transcriptional program markedly distinct from endothelial cells in non-tumoral liver tissue (Figure 4A,B; Figure S5). Strongly upregulated genes included the VEGF receptor *KDR*, the angiocrine factors *SPARC*, *OIT3*, *EMCN*, and *APLNR*, the matrix protein *COL4A1*, and the endothelial activation marker *TM4SF18*, together with *IGFBP7* (Figure 4B). This gene signature defines an endothelial phenotype committed to vascular remodeling, basement membrane deposition, and angiocrine activation [30]. Downregulated transcripts included the cytotoxic lymphocyte markers *GZMK* and *NCAM1*, the T-cell receptor component *CD3E*, and lymphocyte-interaction mediators, indicating silencing of immune-regulatory and leukocyte-interaction programs (Figure 4B).

Pathway enrichment of TEC upregulated genes identified concurrent activation of mitochondrial and structural programs, encompassing mitochondrial protein complex, mitochondrial envelope, and aerobic respiration terms alongside collagen-containing extracellular matrix and external encapsulating structure, indicating metabolic activation of the tumor vasculature in parallel with basement membrane reinforcement (Figure 4C, bottom panel). Downregulated programs were predominantly immune-related, including lymphocyte activation, T cell activation, alpha-beta T cell activation, adaptive immune response, lymphocyte differentiation, and cytosolic ribosome terms, indicating comprehensive silencing of immune-surveillance and translational programs in TECs (Figure 4C, top panel).

The TEC expression program therefore reflects an angiocrine switch: progressive acquisition of pro-angiogenic, matrix remodeling, and oxidative metabolic capacity coupled with enrichment of downregulated gene sets within lymphocyte activation and immune-stimulatory programs. This configuration supports tumor perfusion and vascular expansion while restricting leukocyte adhesion and antigen sampling at the sinusoidal interface, generating immune-exclusionary vascular niches [30].

### 2.7. Intercellular Communication Networks

#### 2.7.1. Consensus Robustness and Network Stability

Intercellular communication was inferred using the LIANA+ consensus framework, integrating CellChat, CellPhoneDB, SingleCellSignalR, and NATMI. The mean Jaccard index across methods increased progressively, reaching a stable plateau at $K^{*}\approx 45$ interactions for HCC donors and $K^{*}\approx 30$ for healthy donors (Jaccard > 0.7), confirming convergence among independent algorithms on a reproducible set of high-confidence interactions (Figure S7) [31]. All downstream analyses were restricted to interactions within this stable consensus range.

#### 2.7.2. Healthy Liver Communication

**Figure 6:**
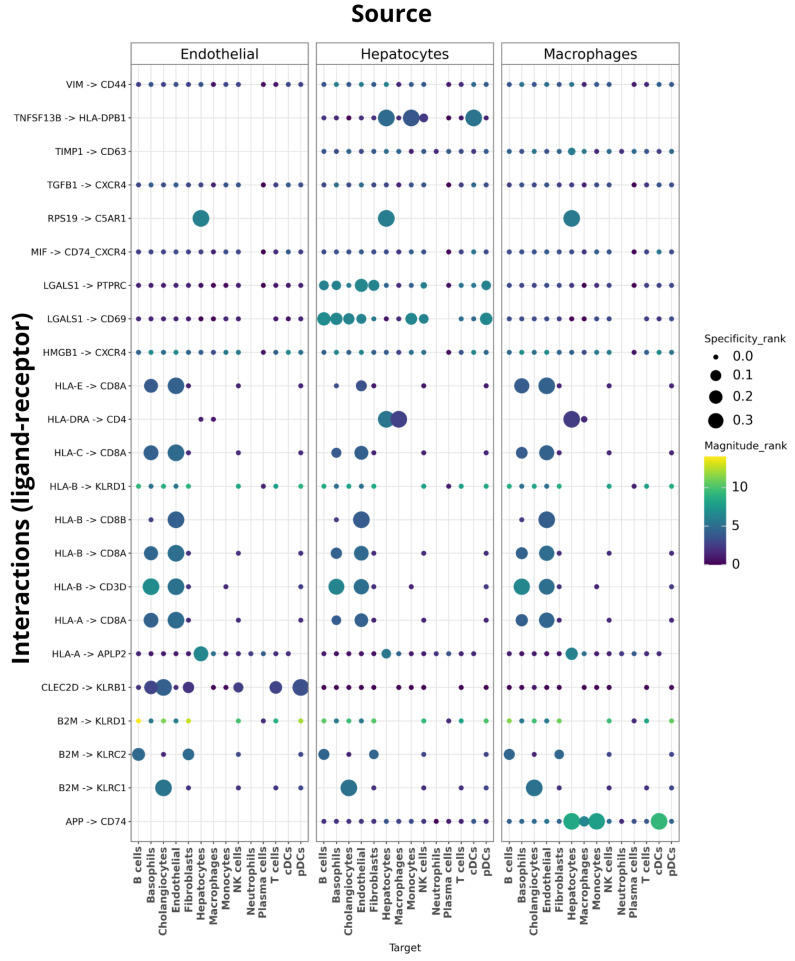
**Consensus-based inference of intercellular signaling networks in healthy liver tissue reveals hepatocytes, endothelial cells, and macrophages as central communication hubs coordinating immune surveillance and tissue homeostasis.** The network is dominated by antigen-presentation pathways, with multiple interactions involving *HLA-A*, *HLA-B*, and *HLA-C* engaging immune receptors such as *CD8A*, *CD4*, and *KLRD1*, consistent with continuous monitoring by cytotoxic T and NK cell populations. Immunomodulatory signaling axes including *MIF–CD74/CXCR4* and *CLEC2D–KLRD1* further suggest coordinated regulation of innate and adaptive immune responses. Galectin-mediated interactions (*LGALS1–SDC1/CD69*) highlight mechanisms supporting immune tolerance, a hallmark of hepatic physiology. Additional pathways involving *HMGB1–CXCR4* and *TGFβ1–CXCR4* indicate basal stress-response and tissue remodeling signals, reflecting the balanced inflammatory control and structural maintenance characteristic of the healthy liver microenvironment.

The healthy liver communication network was dominated by MHC-mediated antigen presentation and innate immune surveillance (Figure 6 and Figure S6) [16,32]. Hepatocytes constituted the principal signal source, driving MHC class I interactions toward CD8^+^ T cells and NK populations via *HLA-A–CD8A*, *HLA-B–CD8A*, *HLA-C–CD8A*, and *HLA-B–CD3D*, supplemented by NK receptor engagements through *B2M–KLRD1*, *B2M–KLRC1*, and *B2M–KLRC2*. Additional hepatocyte-derived interactions, including *LGALS1–CD69*, *LGALS1–PTPRC*, and *RPS19–C5AR1*, reflect modulation of leukocyte activation and complement-responsive myeloid cells, consistent with the immune-buffering role of parenchymal cells [15,33].

Macrophages reinforced antigen presentation to helper T cells through *HLA-DRA–CD4* and directed class I interactions toward CD8^+^ T cells. The *APP–CD74* axis linked macrophages to hepatocytes and monocytes via CD74-associated immune signaling [26], while *MIF–CD74_CXCR4* and *HMGB1–CXCR4* contributed chemokine and danger-signal communication consistent with sentinel functions within the sinusoidal niche [33]. Endothelial cells supplied complementary class I interactions and *CLEC2D–KLRB1* engagement, with *TGFB1–CXCR4* and *VIM–CD44* embedded in programs linking immune modulation to leukocyte interaction at the vascular interface [34]. Collectively, this network reflects the liver’s established role as an immunologically active yet tolerogenic organ: a structured, hepatocyte-centered communication framework that sustains antigen presentation, NK surveillance, and controlled inflammatory tone under homeostatic conditions.

#### 2.7.3. HCC Communication

**Figure 7:**
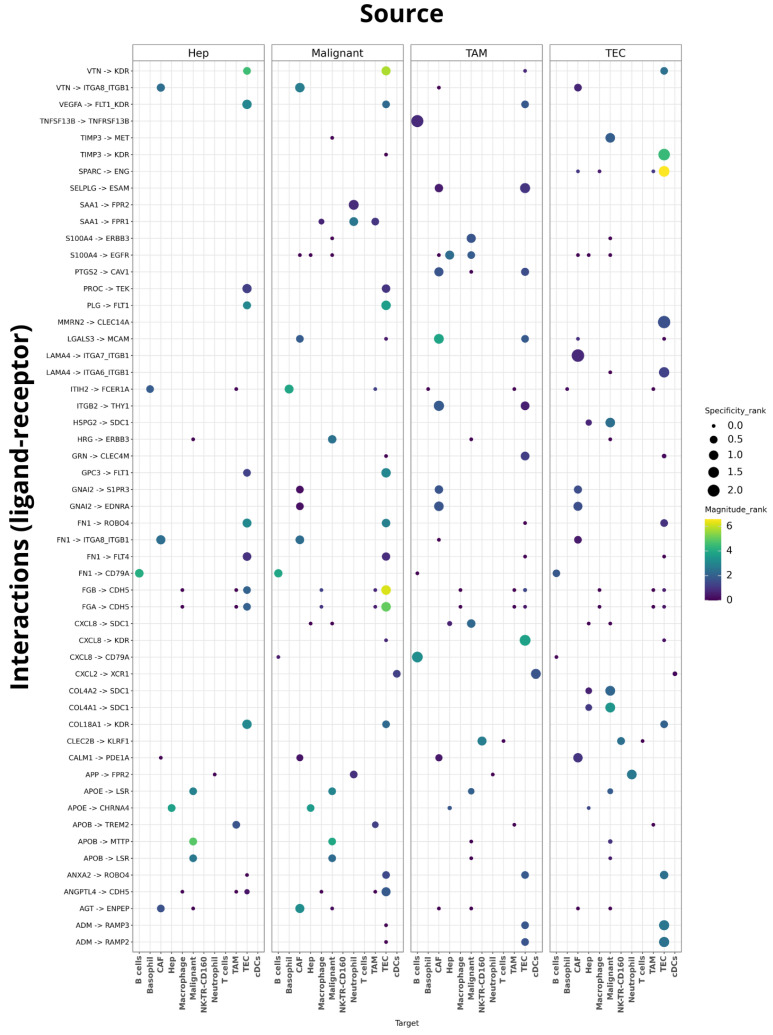
Consensus communication in the HCC microenvironment. The analysis defines a tumor-centered signaling network in which malignant hepatocytes, tumor-associated macrophages (TAMs), and tumor endothelial cells (TECs) act as principal communication hubs sustaining angiogenesis, stromal remodeling, inflammatory recruitment, and metabolic adaptation. Angiocrine interactions such as *VEGFA–KDR* and *PLG–FLT1* reinforce vascular activation, while lipid- and immune-associated axes, including *APOE–LSR* and *APOB–TREM2*, couple metabolic reprogramming with immune modulation. Extracellular matrix–integrin pairs such as *FN1–ITGA8_ITGB1* reflect consolidation of a tumor-supportive signaling architecture.

The HCC microenvironment displayed a qualitatively distinct communication architecture organized around vascular remodeling, ECM restructuring, inflammatory recruitment, and metabolic adaptation, replacing the immune-surveillance-centered logic of the healthy liver (Figure 7) [16].

Malignant hepatocytes engaged endothelial and stromal targets through pro-angiogenic and matrix-dependent interactions: *VEGFA–KDR*, *VTN–KDR*, *COL18A1–KDR*, and *FN1–ITGA8_ITGB1* define sustained endothelial activation and fibronectin–integrin signaling supporting invasion and anchorage plasticity [35,36]. Basement membrane remodeling was further indicated by *COL4A1–SDC1* and *COL4A2–SDC1* interactions, while the lipid-associated axes *APOE–LSR* and *APOB–TREM2* coupled tumor metabolic output with myeloid lipid-sensing pathways [23].

Within the malignant hepatocyte–TEC signaling axis, the LIANA analysis identified *FGB–CDH5* and *FGA–CDH5* interactions as consensus-ranked pairs, pairing fibrinogen subunits β and α with VE-cadherin on tumor endothelial cells. While in vitro biochemical evidence shows that fibrinogen $\beta_{15-42}$ peptide can bind VE-cadherin and disrupt adherens junctions [37,38], the biological significance of this pathway in our HCC dataset requires cautious interpretation. Notably, Kim et al. (2025) [39] demonstrated that FGA and FGB are significantly *downregulated* in bulk HCC tissue relative to healthy liver, with lower fibrinogen expression correlating with worse overall survival and elevated myeloid-derived suppressor cell infiltration. This apparent discordance between their consensus ranking in LIANA and global tissue-level downregulation suggests that these interactions may reflect expression in specific subsets of malignant cells or tumor-adjacent hepatocytes, rather than the dominant bulk malignant population. Furthermore, Han et al. (2024) [40] reported that FGA overexpression in HCC cells *inhibits* cell migration and invasion by activating PI3K/AKT-dependent epithelial marker expression, a phenotype opposed to invasive behavior. Collectively, these considerations indicate that the *FGB/FGA–CDH5* axis, while present in our consensus network, merits cautious interpretation and would benefit from spatial transcriptomic or targeted proteomics validation to determine whether these interactions occur at the tumor–endothelial interface or reflect alternative cellular sources.

TAMs amplified inflammatory and chemotactic circuits. High-confidence interactions, including *CXCL8–SDC1*, *CXCL2–XCR1*, *SAA1–FPR1*, and *SAA1–FPR2*, link macrophage-derived acute-phase signals to leukocyte recruitment and endothelial activation [41]. Growth factor axes *S100A4–EGFR* and *S100A4–ERBB3* connect macrophage-derived inflammatory mediators to epithelial proliferative responses, integrating stromal activation with tumor growth programs [36].

TECs consolidated the angiocrine program: *VEGFA–KDR*, *VTN–KDR*, *COL18A1–KDR*, and *PLG–FLT1* define a persistent vascular activation signature, complemented by adrenomedullin-dependent endothelial survival and barrier adaptation through *ADM–RAMP2* and *ADM–RAMP3* [42]. TEC-derived matrix interactions and syndecan-linked chemokine signaling further shaped immune-cell trafficking within perivascular niches.

The transition from homeostatic to tumoral communication reflects qualitative rewiring rather than quantitative amplification alone. Whereas healthy liver signaling sustains immunological equilibrium within a zonated metabolic framework, the HCC network displaces antigen-presentation circuits with pro-angiogenic, ECM–integrin, chemokine, and lipid-associated modules [15,16]. Malignant hepatocytes, TAMs, and TECs collectively assume the dominant signaling burden: malignant hepatocytes instruct endothelial and stromal remodeling, TAMs amplify inflammatory and matrix turnover signals, and TECs reinforce vascular expansion. Residual homeostatic hepatocyte–immune exchanges are comparatively attenuated, indicating partial erosion of the antigen-presentation-oriented framework that defines normal liver physiology [9,16].

### 2.8. Cross-Validation Performance Across Diseased and Healthy Cohorts

Cross-validation evaluated the robustness of the automated annotation framework across both study cohorts, assessing generalization under tumor heterogeneity and physiological conditions (full tables and figures for all of the analyses in this section can be found in the cross_validation_analysis folder within the Supplementary Materials). The framework demonstrated consistent performance in both settings, with F1 scores exceeding 0.85 across folds and standard deviations below 0.02, confirming reproducible predictions under repeated resampling (see consensus global metrics: DiseasedCV_consensus_global_ metrics_mean_sd.csv; HealthyCV_consensus_global_metrics_mean_sd.csv). Close agreement between macro- and micro-averaged scores indicated balanced classification across both abundant and minority cell populations (global metrics by fold: DiseasedCV_global_metrics_by_fold.csv; HealthyCV_global_metrics_by_fold.csv).

In HCC samples, hepatocytes and malignant hepatocytes achieved the most stable classification, with per-class F1 scores frequently exceeding 0.95 (Figure S1). T cells demonstrated robust performance, although variability increased when distinguishing proliferative or effector subsets, reflecting the transcriptional overlap inherent to activated T-cell states within the tumor. TAMs and endothelial cells reached intermediate precision and recall values with greater fold-to-fold variability (Table S2), consistent with their documented heterogeneity in HCC. Rare populations, including mast cells and proliferative T cells, showed lower classification stability, driven by limited patient-level representation (Figure S2; DiseasedCV_per_class_consensus.csv).

In healthy donors, hepatocytes and cholangiocytes achieved classification metrics approaching unity across folds (Figure S3). Endothelial cells and B cells were robustly classified with slightly higher variability, while T cells showed intermediate performance, reflecting transcriptional plasticity even under physiological conditions. Rare populations, including plasmacytoid dendritic cells and mast cells, produced the lowest and most variable scores in both cohorts, attributable to training data scarcity (Figure S4; HealthyCV_per_class_consensus.csv).

Posterior probability distributions corroborated these trends. Major parenchymal and stromal populations exhibited sharply peaked distributions with medians above 0.9 and narrow interquartile ranges (Figures S1 and S3), indicating high prediction confidence. Immune subsets—macrophages and regulatory T cells—displayed broader distributions (Figures S2 and S4), consistent with transcriptional intermediacy and context-dependent states. Low-confidence predictions (posterior probability < 0.5) represented fewer than 5% of cells in HCC cohorts and fewer than 7% in healthy donors, restricted predominantly to ambiguous boundaries between immune subtypes (DiseasedCV_pred_score_by_class_summary.csv; HealthyCV_pred_score_by_class_summary.csv).

Average precision values corroborated population-level trends: hepatocytes, malignant hepatocytes, and T cells consistently exceeded 0.95 in diseased cohorts (Figure S2), while macrophages, endothelial cells, and B cells occupied the 0.80–0.90 range. Healthy donors showed a comparable distribution, with hepatocytes, cholangiocytes, and endothelial cells above 0.90 and immune populations between 0.80 and 0.88. The stability of these distributions across folds confirms that the observed performance reflects reproducible model behavior rather than artifacts of data partitioning (DiseasedCV_average_precision _consensus.csv; HealthyCV_average_precision_consensus.csv).

## 3. Discussion

The central finding of this study is that HCC progression operates as a multicellular ecological process: malignant hepatocytes, TAMs, and TECs do not acquire pro-tumorigenic properties independently but converge through structured ligand–receptor circuits into a self-sustaining network that collectively enforces metabolic adaptation, immune exclusion, and vascular remodeling. This systems-level view extends beyond cataloging cell-type alterations to demonstrate that the functional architecture of the TME is an emergent property of intercellular cooperation rather than the sum of isolated transcriptional changes [15,43,44].

A defining feature of this cooperation is the coupling between metabolic reprogramming and communication rewiring. Prior single-cell studies have characterized immunosuppression and angiogenesis as partially independent phenomena in HCC [16,32]. This study provides evidence that these processes are mechanistically linked through the same intercellular signaling circuits: the lipid-associated axes *APOE–LSR* and *APOB–TREM2* bridge hepatocyte metabolic output to myeloid immune polarization, pro-angiogenic signals from malignant hepatocytes (*VEGFA–KDR*, *COL18A1–KDR*) directly instruct endothelial remodeling, and TAM-derived inflammatory chemokines (*CXCL8–SDC1*, *SAA1–FPR1/2*) feed back to amplify vascular activation. Immunometabolic reprogramming in HCC is therefore not a byproduct of malignant growth; it is structurally encoded in the communication network.

Malignant hepatocytes displayed upregulation of gene sets encoding both glycolytic enzymes and mitochondrial oxidative phosphorylation (OXPHOS) complexes, a transcriptional pattern consistent with metabolic plasticity at the gene expression level. We emphasize that this pattern represents transcriptional capacity rather than direct measurement of metabolic flux; actual pyruvate oxidation rates, glucose consumption, and ATP production would require complementary functional assays such as Seahorse analysis or isotopic tracing [45]. Nonetheless, the coexistence of upregulated glycolytic and OXPHOS gene signatures is consistent with conceptual models wherein HCC cells maintain multiple catabolic pathways to sustain proliferation under varying oxygen and nutrient availability [46,47]. Transcriptionally, malignant hepatocytes simultaneously downregulated MHC class I loading components and immune-sensing genes, creating a pattern wherein metabolic and immune-evasive gene sets are concurrently dysregulated. Whether this coordinateness reflects mechanistic coupling (e.g., through hypoxia or lactate signaling) or independent selective pressures cannot be determined from transcriptional data alone [23,48]. Metabolic transcriptional reprogramming thus represents an important organizing feature of the HCC phenotype that generates downstream signals affecting surrounding immune and stromal compartments, although the extent to which transcriptional markers predict actual metabolic activity warrants validation through functional metabolomics and spatial analysis.

TAMs reinforced this immunometabolic niche through complementary programs. The *TREM2*-enriched, lipid-scavenging macrophage phenotype observed here is consistent with a PPAR-driven M2 polarization reported in human and murine HCC datasets [17,26,49]. APOE-linked lipoprotein signals from malignant hepatocytes sustain this immunosuppressive macrophage state, coupling tumor lipid output with myeloid reprogramming [17,50]. Downregulation of mitochondrial respiratory programs in TAMs indicates metabolic reconfiguration away from oxidative self-sufficiency, likely supporting the lipid-handling and secretory functions that define their pro-tumorigenic role. Concurrently, *SPP1–CD44* signaling drives ECM remodeling and stromal restructuring, reinforcing niches for malignant and cancer stem-like populations [50,51].

TECs underwent a parallel angiocrine switch: upregulation of *VEGFA*, *ANGPT2*, and *KDR* family signaling, combined with attenuation of interferon and TLR pathways, generated a vasculature that simultaneously supports tumor perfusion and restricts immune infiltration [52,53]. The resulting vascular niches impede the access of T cells and dendritic cells to the tumor parenchyma, while enabling metabolic exchange and stromal conditioning. Through reinforced *VEGFA–KDR* and *ADM–RAMP2/3* circuits, TECs and TAMs establish reciprocal feedback that stabilizes the malignant ecosystem [15,35].

At the tissue scale, this immunometabolic remodeling is encoded in the rewiring of intercellular communication. In healthy liver tissue, hepatocytes drive a parenchyma-centered network of antigen presentation and immune surveillance, coordinating homeostasis through MHC–TCR interactions and complement-responsive circuits [41]. In HCC, this architecture is replaced by a tumor-driven network in which angiogenic signals (*VEGFA–KDR*, *PLG–FLT1*), ECM–integrin interactions (*FN1–ITGA8_ITGB1*, *COL4A1–SDC1*), inflammatory chemokines (*CXCL8–SDC1*, *CXCL2–XCR1*), and lipid-associated axes (*APOB–TREM2*, *APOE–LSR*) collectively displace immune-regulatory exchange [15,52]. Matrix proteolysis driven by ADAM and MMP family members further generates invasion-permissive structural niches, while *CCL2–CCR2* and *SPP1–CD44* couple myeloid recruitment to niche construction [17,36].

These cooperative signaling dependencies carry direct therapeutic implications that extend well beyond current standard-of-care regimens. At the level of gene expression, malignant hepatocytes display upregulation of both glycolytic and oxidative metabolic genes—a pattern that confers apparent resilience to single-pathway metabolic inhibition, as cells retain the capacity to reroute fluxes between cytosolic and mitochondrial compartments to sustain ATP and biosynthetic precursors [46,47]. Combinatorial metabolic targeting—pairing glycolytic blockade (2-deoxy-D-glucose or LDHA inhibitors) with OXPHOS inhibition (e.g., IACS-010759, a mitochondrial complex I inhibitor under clinical evaluation in advanced solid tumors)—may be required to collapse this transcriptional flexibility and sensitize hepatocytes to downstream immune-mediated killing [47].

At the stromal level, the interdependence of TREM2-enriched myeloid immunosuppression, lipid-driven TAM reprogramming, and angiocrine TEC programs argues for strategies that disrupt intercellular cooperation rather than targeting individual intracellular nodes [53,54]. The TREM2^+^ immunosuppressive TAM subset identified here is supported by preclinical evidence as a potential therapeutic target: TREM2 blockade has been shown to reinvigorate cytotoxic T-cell infiltration and synergize with PD-1 checkpoint inhibitors in murine HCC models [55], and CD36 inhibition—which interrupts lipid uptake in myeloid cells in vitro—offers a complementary metabolic approach currently undergoing evaluation [54]. However, translation of these preclinical findings to clinical benefit in human HCC requires formal clinical trial validation.

The *VEGFA–KDR* angiogenic axis that we detected in TEC–hepatocyte interactions maps directly onto the molecular target of approved HCC therapies: sorafenib and lenvatinib suppress VEGFR kinase activity, while the bevacizumab–atezolizumab combination simultaneously blocks VEGF-A ligand and restores T-cell function, mechanistically addressing both the angiogenic program and the immune exclusion that we observe in TEC–lymphocyte interactions [53,56].

*CSC-high* hepatocytes with activated Wnt/Notch/Hedgehog-related transcriptional signatures are a plausible target for Hedgehog pathway inhibitors such as vismodegib, which have reached clinical testing in solid tumors, and for Wnt pathway antagonists currently in early-phase trials in HCC [57]. The enrichment of *FTH1* in CSC-high hepatocytes connects the cancer stem-cell state to ferroptosis resistance, since FTH1-mediated iron storage protects against lipid peroxidation. Pharmacological ferroptosis inducers, including the GPX4 inhibitor RSL3 and the pro-ferroptotic mechanism of sorafenib itself, could thus selectively destabilize this subpopulation [58].

The *FGB/FGA–CDH5* endothelial permeability axis identified by LIANA constitutes an underexplored target at the malignant hepatocyte–TEC interface; disrupting fibrinogen-mediated VE-cadherin destabilization could reinforce the endothelial barrier, attenuate intravasation, and reduce MDSC recruitment, potentially complementing anti-angiogenic therapies that converge on the same vascular node. Preclinical and early clinical evidence supports this multi-node reasoning: combining anti-angiogenic and immune-modulatory therapies reprograms the vasculature and enhances immune infiltration, addressing two interconnected nodes of the malignant circuit simultaneously [15,53], and nivolumab- or pembrolizumab-based regimens are now actively evaluated in combination with VEGFR inhibitors specifically to exploit the immunometabolic vulnerabilities that scRNA-seq analyses like the present study are beginning to map at single-cell resolution.

Defining HCC tumors by the relative dominance of these cooperative modules—metabolic, immunoregulatory, and angiogenic—offers a practical framework for patient stratification and rational therapy selection [57,59]. Quantitative intercellular network scores summarizing ligand–receptor flux, chemokine recruitment intensity, and metabolic cross-feeding between defined populations could serve as predictive indices for therapeutic response, analogous to transcriptional predictors already under clinical evaluation [15].

The LIANA consensus analysis identified *FGB–CDH5* and *FGA–CDH5* as ranked interaction pairs within the malignant hepatocyte–TEC signaling axis (Figure S5). In vitro biochemical studies demonstrate that fibrinogen beta and alpha subunits can bind VE-cadherin through the fibrinogen $\beta_{15-42}$ fragment and disrupt endothelial junctional integrity in cell culture systems [37,38]. However, interpreting these interactions within the context of our HCC dataset requires consideration of conflicting evidence.

Kim et al. (2025) [39] demonstrated that FGA and FGB are *broadly downregulated* in HCC tumor tissue relative to healthy liver tissue, with reduced fibrinogen expression correlating with worse overall survival and elevated myeloid-derived suppressor cell abundance—a pattern that appears discordant with a role for fibrinogen as a dominant pro-invasive ligand in the bulk malignant population. This discrepancy may reflect differential expression patterns across tumor subregions, tumor-adjacent tissue, or specific transcriptional states (Figure S5); indeed, we observed FGB among the top markers of CSC-high cells, suggesting possible concentration in this subpopulation.

Additionally, Han et al. (2024) [40] reported that FGA overexpression in HCC cells *suppresses* migration and invasion through PI3K/AKT-dependent restoration of epithelial E-cadherin, a phenotype opposing invasive behavior. Collectively, these considerations suggest that the *FGB/FGA–CDH5* interactions identified by LIANA warrant cautious interpretation: they may reflect spatially restricted expression in CSC-high populations or tumor-adjacent cells, rather than a dominant mechanism operating in the bulk malignant epithelium. Spatial transcriptomics or high-resolution multiplexed immunofluorescence would be needed to determine whether these ligand–receptor pairs actually engage at the tumor–endothelial interface, as well as which cell compartments mediate their expression.

These interpretations are constrained by inherent limitations of the data. The datasets are cross-sectional and enriched for advanced lesions, precluding direct inference of temporal progression or early oncogenic events [60]. Transcriptomic profiling captures mRNA abundance but not protein activity, post-translational modifications, or metabolic flux, requiring complementary functional validation [45,61].

A particularly important methodological limitation concerns the metabolic inferences drawn from gene expression data. Throughout this study, metabolic reprogramming was characterized by differential expression of genes encoding metabolic enzymes, transporters, and regulators—a transcriptional proxy that reflects the cell’s biosynthetic and energetic potential rather than its instantaneous metabolic activity. Gene-level surrogates for glycolysis (e.g., *LDHA*, *PKM*, *SLC2A1*), OXPHOS (e.g., *NDUFA1*, *COX7C*, *ATP5F1A*), and lipid metabolism do not directly measure metabolite concentrations, enzymatic rates, or isotopic flux distributions. The discordance between transcript abundance and actual metabolic flux has been documented across cancer contexts and validated only in select instances for HCC [45,62]. Consequently, the upregulation of both glycolytic and oxidative gene sets in malignant hepatocytes, the reduced OXPHOS gene expression in CSC-high cells, and the lipid-sensing axes involving TREM2 and APOE should all be interpreted as transcriptional patterns and tendencies rather than confirmed metabolic flux states. Direct functional measurements via Seahorse assays, isotopic tracing, or metabolomic profiling would be required to validate whether transcriptional patterns predict actual glucose consumption, ATP production, or lipid metabolism rates.

Cross-validation with spatial or metabolomic modalities was not feasible in the present study because the five publicly deposited datasets that we analyzed (GEO: GSE242889, GSE228195, GSE151530, GSE189903; CZ CELLxGENE) do not include matched spatial transcriptomics or metabolomics measurements. Spatially resolved data would be particularly valuable for two reasons: First, hepatic zonation, the physiological gradient of metabolic gene expression from periportal to pericentral zones, could confound cluster-level metabolic comparisons unless tissue coordinates are available; Guilliams et al. (2022) [63] demonstrated that ignoring spatial context masks macrophage niche heterogeneity in the liver. Second, spatial metabolomics in liver tissue has revealed that metabolite distributions often do not co-localize with the transcriptional domains predicted from scRNA-seq, underscoring the need for direct measurement [64]. For intercellular communication, spatial resolution would allow discrimination of juxtacrine from paracrine interactions and confirm whether high-ranked LIANA pairs reflect physically proximal cell populations, as illustrated by resistance mechanisms to HCC immunotherapy that were only resolved through spatial transcriptomics [61]. Multimodal integration, coupling spatial transcriptomics with targeted metabolomics and single-cell proteomics, represents the most direct path to confirming that the transcriptional circuits described here correspond to active biochemical processes and physical cell–cell contact in the tumor tissue [32,64].

The reproducibility of the core patterns described here—coordinated metabolic activation, myeloid immunosuppression, and endothelial angiocrine remodeling—across independent cohorts in multiregional studies supports their biological robustness [17,43,60]. Incorporating module-level scores into existing HCC molecular classifications and developing spatially informed indices of ecosystem interdependence would represent concrete next steps toward mechanism-based therapeutic design that treats the tumor as a coordinated multicellular system [32,53,59,62].

The identification of CSC-like subpopulations within the malignant compartment adds an important dimension to the immunometabolic model proposed here. CSC-high cells, defined by a composite signature from Lin et al. (2024) [24] and Ding et al. (2025) [25], constituted approximately 30% of malignant cells and displayed a transcriptionally distinct phenotype characterized by reduced OXPHOS activity, suppression of immune effector genes, and activation of Wnt and Hedgehog self-renewal pathways. The predominance of *FTH1* among CSC-high markers is noteworthy: FTH1-mediated iron sequestration has been linked to ferroptosis resistance and stemness maintenance in hepatic malignancy [24], suggesting that iron metabolism may constitute a convergence point between stemness and immune-evasion programs. These findings are consistent with the broader model of immune exclusion described for the HCC TME: CSC-high cells appear to extend immune silencing beyond the bulk malignant compartment into the stem-like subpopulation. Unlike Lin et al. (2024) [24], who identified CSC-mediated crosstalk primarily through MIF–CD74/CXCR4 and MDK–NCL axes, our dataset does not include sufficient CSC-specific LIANA resolution to definitively assign ligand–receptor pairs to the CSC-high subpopulation, representing a limitation that future spatially resolved analyses could address. The molecular heterogeneity within the malignant compartment, along with the presence of a stem-like resistant subpopulation, underscores the importance of considering intratumoral diversity when designing therapeutic strategies that target the HCC microenvironment.

## 4. Materials and Methods

### 4.1. Data Sources and Processing

To explore the immunometabolic reprogramming and cell–cell communication within the hepatocellular carcinoma (HCC) microenvironment, we utilized publicly available single-cell RNA sequencing (scRNA-seq) datasets from the Gene Expression Omnibus (GEO) database. Specifically, we retrieved tumor-derived scRNA-seq data from [17,65], which included samples from patients diagnosed with advanced-stage HCC. For healthy liver comparisons, we incorporated high-quality datasets from [13,66,67,68], which together provided a robust baseline of cellular profiles from 4 healthy donors with histologically confirmed non-cancerous liver tissue. All datasets were generated using high-throughput droplet-based sequencing platforms (10x Genomics Chromium; 10x Genomics, Inc., Pleasanton, CA, USA), ensuring consistency in data type and quality across studies. The overall analysis workflow is summarized in Figure 1.

The combined tumor dataset consisted of 47,601 cells derived from multiple regions of primary HCC tumors, while the healthy liver dataset comprised 52,261 cells sampled from histologically confirmed non-cancerous tissues from 4 healthy donors. The selection of datasets emphasized both spatial diversity and cellular representation to minimize biases associated with sampling location or sequencing depth. Raw count matrices and metadata were downloaded in h5ad or Matrix Market formats and loaded into an AnnData object using the Scanpy library (v1.9.3) [69] for preprocessing and downstream analysis.

Upon loading the data, all gene identifiers were mapped to a standardized nomenclature (HGNC symbols), and mitochondrial genes were flagged based on the presence of the “MT-” prefix. To ensure uniformity, the datasets were filtered to retain only protein-coding genes, and ribosomal genes were excluded from downstream normalization to avoid artifacts associated with highly expressed housekeeping transcripts. Metadata from original publications, including patient ID, diagnostic condition, and sequencing batch, were integrated into the AnnData object to allow for batch-aware normalization and stratified analysis.

To facilitate cross-sample integration, we applied batch correction and joint embedding using the scVI (scvi-tools v1.3.3; https://scvi-tools.org/) algorithm [70], allowing us to align cells across donors and conditions while preserving biological variability. Batch effects, which often arise due to technical differences in tissue dissociation, library preparation, or sequencing, were evaluated and visualized using principal component analysis (PCA) and uniform manifold approximation and projection (UMAP) before and after correction (Figure 2).

**Figure 8:**
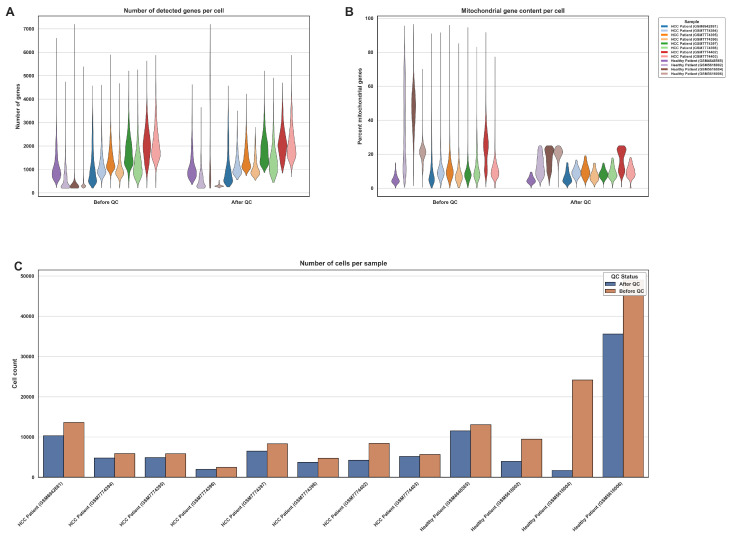
**Quality control metrics:** (**A**) Distribution of the number of genes detected per cell across samples before and after quality control filtering. (**B**) Proportion of mitochondrial gene expression per cell, used to assess cell integrity. Cells with high mitochondrial content were excluded. (**C**) Total number of cells retained per sample following initial filtering based on gene counts and mitochondrial content.

Quality control filtering was conducted to eliminate low-quality cells and potential sequencing artifacts. Cells were retained if they met the following thresholds: (1) expression of >200 and <7500 unique genes per cell, (2) <25% mitochondrial gene content, and (3) <10% ribosomal gene content. Outlier detection was additionally performed using median absolute deviation (MAD) statistics across multiple QC parameters, and cells falling beyond ±3–5 MADs from the median were excluded (Figure 8). The 25% mitochondrial content threshold was selected after empirical evaluation of the score distribution across all samples. Applying more stringent cut-offs (15–20%) excluded >50% of the total cell population, disproportionately removing hepatocytes—a cell type known to maintain constitutively elevated mitochondrial transcript proportions due to their high metabolic activity [71]. The 25% threshold aligns with values reported in comparable human liver single-cell studies: Payen et al. (2021) [72] applied ≤25% for hepatic stellate cell profiling in human liver, Meng et al. (2025) [73] used <25% for hepatocyte and cholangiocyte analysis, and Wen et al. (2022) [74] applied <20% for single-cell and single-nucleus HCC comparisons. Notably, MacParland et al. (2018) [71] evaluated six mitochondrial cut-offs spanning 10–60% in human liver tissue and found that all major cell populations were robustly recovered across thresholds, while hepatocytes were most susceptible to loss under stricter filtering; those authors ultimately adopted 50% to preserve hepatocyte representation. Our choice of 25% therefore represents a conservative intermediate that balances removal of damaged or lysed cells against preservation of metabolically active hepatocyte populations.

Following quality control, we performed library size normalization to correct for differences in sequencing depth. Gene counts for each cell were normalized to a total of 10,000 transcripts and log-transformed using a pseudocount of 1. Highly variable genes (HVGs) were selected using Scanpy’s built-in dispersion-based method, identifying the top 2000 genes with the greatest normalized variance across cells. These HVGs were subsequently used for PCA (*n* =50 components), which formed the basis for neighborhood graph construction, UMAP projection, and downstream clustering.

In total, the integrated and quality-controlled dataset retained 93,032 high-quality single-cell transcriptomes—40,771 from HCC tumor-derived samples and 52,261 from 4 healthy donors—spanning a diverse array of cell types, including parenchymal hepatocytes, immune cell populations, endothelial cells, fibroblasts, and cholangiocytes (Figure 8). Quality control filtering removed low-quality cells based on gene count thresholds, mitochondrial content, and ribosomal contamination, ensuring robust downstream analyses. This high-resolution cellular atlas provided the foundation for subsequent annotation, differential expression, pathway enrichment, and ligand–receptor interaction analyses aimed at unraveling the molecular architecture of the HCC tumor microenvironment.

#### 4.1.1. Doublet Removal

Doublet detection and removal represent a critical preprocessing step in single-cell RNA sequencing (scRNA-seq) data analysis. During the encapsulation of single cells into microdroplets—particularly in high-throughput droplet-based platforms like 10x Genomics Chromium—there is a non-negligible probability that two or more cells are co-encapsulated into the same droplet. These multiplets, or *doublets*, result in hybrid transcriptional profiles that do not correspond to any true biological cell type. If left unaddressed, doublets can significantly skew downstream analyses, such as cell-type identification, differential gene expression, and trajectory inference, potentially introducing false-positive cell clusters and misleading biological conclusions.

To mitigate this artifact, we employed the Solo algorithm: a state-of-the-art, deep learning-based tool for doublet detection specifically designed for scRNA-seq data [75]. Solo is built upon scVI (single-cell variational inference), a variational autoencoder framework that learns a nonlinear latent representation of the gene expression space. By leveraging this latent space, Solo simulates artificial doublets and trains a classifier to distinguish real cells from doublets based on complex expression patterns. Unlike traditional methods such as Scrublet or DoubletFinder, Solo uses a probabilistic approach that adapts to the nonlinear and high-dimensional structure of real scRNA-seq data, yielding higher accuracy, particularly in complex tissues with broad cellular heterogeneity—such as the HCC tumor microenvironment.

The doublet removal workflow began with loading raw count matrices into an AnnData object and performing initial quality control to exclude low-quality or outlier cells that might interfere with doublet modeling. We filtered out genes with extremely low expression, retaining only those present in at least 10 cells. Using the default parameters, we trained the Solo model for 400 epochs using a GPU-accelerated environment with CUDA-enabled support to optimize computational efficiency. This training allowed the model to converge on a well-structured latent space and to robustly identify doublets based on their deviation from biologically plausible gene expression profiles.

The detection phase involved simulating synthetic doublets by averaging the expression profiles of random cell pairs and introducing them into the dataset. The model then classified both real and synthetic cells, assigning a posterior probability of being a doublet to each observed cell. To ensure high-confidence retention, we kept only cells with a posterior singlet probability of ≥0.90, thereby restricting downstream analyses to cells most reliably classified as singlets. This threshold was chosen based on the distribution of scores and cross-validation with known cell-type boundaries, aiming to balance sensitivity (detecting true doublets) and specificity (retaining real single cells).

To evaluate the performance of Solo in our datasets, we examined the doublet score distributions across different cell types and donors. We also assessed doublet localization in UMAP embeddings: genuine doublets often localize between two distinct cell populations in reduced dimensional space. The removal of these putative doublets improved the sharpness of cluster boundaries and enhanced the interpretability of downstream cell-type classification and trajectory modeling.

In total, Solo identified and filtered out approximately 4.1% of cells in the HCC dataset and 3.7% in the healthy liver dataset (full model parameters and per-dataset performance metrics are reported in Supplementary Table S1). These proportions are consistent with the expected doublet rates for 10x Genomics experiments at the observed cell-loading densities. The resulting clean datasets provided a more accurate and biologically faithful representation of the cellular composition in both tumor and non-tumor liver tissues, serving as a robust foundation for subsequent analyses such as differential expression, gene regulatory network inference, and intercellular communication modeling.

#### 4.1.2. Cell-Type Annotation

Accurate cell-type annotation is a cornerstone of single-cell RNA sequencing (scRNA-seq) studies, as it enables researchers to decipher the cellular composition, heterogeneity, and functional organization of tissues under physiological and pathological conditions. In the context of hepatocellular carcinoma (HCC), where both the tumor and its microenvironment comprise a diverse mixture of epithelial, immune, endothelial, and stromal cells, high-resolution annotation is essential to disentangle tumor-specific signatures from surrounding non-malignant cell types and to identify rare or transient cell states that may be critical for disease progression or therapeutic resistance [9].

To achieve robust and context-specific annotation of the 93,032 cells in our integrated dataset (tumor and healthy liver combined), we implemented a multi-tiered strategy that combined automated machine learning classifiers with probabilistic modeling and manual expert validation. This ensemble approach mitigates common limitations of single-method annotation pipelines, such as overreliance on reference datasets or inability to resolve ambiguous or novel cell states.

##### Automated Annotation Using CellTypist

As a first step, we applied CellTypist (https://www.celltypist.org/), a supervised machine learning-based classifier tailored for scRNA-seq data. CellTypist (v1.7.1; https://www.celltypist.org/) uses logistic regression or stochastic gradient descent classifiers (XGBoost via scikit-learn v1.7.2) trained on manually curated reference atlases to assign cell-type labels based on transcriptomic profiles. For this study, we constructed and used two classes of CellTypist models:

1. Tumor-specific models were trained using annotated scRNA-seq datasets from the NCI-CLARITY consortium [76], the Multi-Regional HCC Atlas [77], and the Sequential HCC Atlas [78]. These models capture both canonical and disease-specific cell types, such as malignant hepatocytes, cholangiocarcinoma-like cells, tumor-associated macrophages (TAMs), cancer-associated fibroblasts (CAFs), and tumor endothelial cells (TECs).
2. Healthy liver models were derived from public datasets [63] and the CellxGene liver cell atlas, containing over 160,000 transcriptomes from non-malignant human liver tissues. These models were optimized to detect healthy hepatocytes, Kupffer cells, hepatic stellate cells, cholangiocytes, sinusoidal endothelial cells, and resident innate lymphoid cells.

CellTypist was run with default preprocessing parameters and included confidence scoring for each prediction. Labels with low confidence (<0.5 probability) were flagged for additional inspection. This dual-model strategy allowed us to compare label concordance across tumor and non-tumor conditions, and to identify condition-specific expression shifts within shared cell types (e.g., naïve vs. exhausted T cells, resting vs. activated stellate cells).

##### Label Refinement Using scANVI

To further enhance annotation robustness and to resolve uncertain or transitional cell states, we applied scANVI (single-cell annotation via variational inference), a semi-supervised extension of the scVI probabilistic framework [79]. Unlike deterministic classifiers, scANVI models cell identity as a distribution over possible types, which is particularly advantageous for tissues undergoing dynamic changes such as inflammation, regeneration, or malignant transformation.

The scANVI model was first trained in an unsupervised fashion on the full gene expression matrix using highly variable genes, thereby learning a latent representation that captures the biological structure of the data. Then, using high-confidence CellTypist annotations as anchor labels, scANVI inferred probabilistic labels for all cells—especially for those in ambiguous clusters, at tumor–non-tumor interfaces, or representing rare subtypes. This label transfer approach allowed for cross-condition generalization, enabling meaningful comparisons between the tumor and healthy compartments (Figure 3).

### 4.2. Clustering Optimization with Adjusted Rand Index (ARI)

We optimized the clustering resolution of our single-cell datasets to avoid both under- and over-segmentation of cell populations. To this end, we used our scVI model, which served as input for pseudoclustering. A grid search over multiple neighborhood sizes (*k*), distance metrics, and Leiden resolutions was conducted, and for each parameter set, clustering results were compared to preliminary annotations obtained from scANVI using the Adjusted Rand Index (ARI). The ARI provides a measure of concordance between two partitions, correcting for chance agreement, and is widely applied in cluster validation. Silhouette scores were additionally computed on a random subset of cells to assess cluster cohesion and separation, serving as a secondary criterion in case of ties.

For each dataset, the configuration that maximized the ARI (and silhouette score in case of ties) was selected as the optimal resolution, and the resulting cluster assignments were stored as a new column in Anndata objects. To further assess the robustness of this solution, ARI values were calculated separately per batch and per patient sample, and cluster purity scores relative to scANVI-derived labels were reported. The optimal clustering was then transferred to the main AnnData object, and each cluster was relabeled according to its majority annotation and manual inspection of marker gene expression, ensuring biologically coherent designations.

This ARI-guided clustering strategy was the basis for defining the granularity of cellular partitions, including cross-validation, differential expression, and ligand–receptor inference. By anchoring clustering to both reference-informed labels and intrinsic transcriptomic structure, it is possible to minimize risks of spurious subdivisions and preserve resolution sufficient to capture relevant cellular heterogeneity.

#### 4.2.1. Manual Curation and Marker-Based Validation

In addition to the utilization of automatic annotation and ARI-based clustering optimization, we conducted a comprehensive manual validation phase. For each putative cell cluster, we did the following:

1. Calculated expression scores for known marker gene sets derived from PanglaoDB, CellMarker, and recent liver-specific single-cell atlases from CELLxGENE.
2. Applied Wilcoxon rank-sum tests to identify the top differentially expressed genes for each cluster and compared them against reference signatures.
3. Visualized gene expression patterns using dotplots, violin plots, and feature plots to verify consistency with expected cell phenotypes.
4. Relabeled or merged clusters as needed, in the event of unclear lineage identity or putative doublet contamination.

#### 4.2.2. Post Hoc Marker Validation and Refinement

To further ensure annotation robustness and identify any residual mislabeling, we implemented a systematic post hoc validation strategy based on canonical marker gene expression. For each of the 19 major cell types represented in the dataset, we compiled evidence-based marker gene sets from PanglaoDB, CellMarker, and recent liver-specific single-cell atlases. For each cell, we computed an expression score for each cell-type marker set using the Seurat method and applied a *winner-takes-all* reclassification approach: cells were reclassified to the cell type with the highest marker score if that score exceeded a predefined confidence threshold (hepatocyte threshold = 0.50; all other cell types = 0.30), following the scoring implementation in Seurat [80]. This threshold strategy recognizes that hepatocyte-specific markers (e.g., ALB, APOA1, TTR) are exceptionally robust and specific, while other markers require lower stringency due to broader biological contexts. Cells in which no marker set exceeded the threshold retained their original scANVI assignment. This refined annotation strategy identified and corrected systematic mislabelings introduced during reference-based transfer, ensuring that all reported cell compositions and subsequent analyses reflected biologically validated cellular identities.

The complete canonical marker gene panel used for validation is documented in Supplementary Table S3 (Canonical Cell Type Markers), enabling transparency and allowing readers to assess the biological basis for cell-type assignments throughout this study.

#### 4.2.3. Outcome of Annotation Workflow

Major annotated types included malignant hepatocytes, immune subtypes (T cells, B cells, TAMs, dendritic cells, NK cells), hepatic parenchymal and stromal cells (hepatocytes, Kupffer cells), and vascular components (arterial/venous endothelial cells, TECs) (Figure 3C). Several subclusters also emerged, including proliferative malignant cells and inflammation-associated fibroblasts—each with unique expression profiles and potential biological relevance.

This high-resolution annotation provided the foundation for all subsequent analyses, including cell-type-specific differential expression, metabolic pathway inference, and intercellular communication modeling.

### 4.3. Differential Gene Expression and Pathway Enrichment

Differential gene expression (DGE) analysis is central to understanding the molecular mechanisms that distinguish malignant and non-malignant cellular states. In the context of single-cell RNA sequencing (scRNA-seq), DGE enables the identification of cell-type-specific transcriptional programs associated with disease processes such as oncogenic transformation, immune modulation, and metabolic reprogramming. However, the inherent sparsity and technical noise in single-cell data demand the use of statistical methods capable of modeling low and overdispersed count distributions with high accuracy.

For this study, we performed DGE analysis using the edgeR package, a well-established tool that implements empirical Bayes estimation within a negative binomial framework [81,82]. Although initially developed for bulk RNA-seq, edgeR has been successfully adapted to scRNA-seq data—particularly in pseudo-bulk configurations, where expression counts from cells of the same type and condition are aggregated to reduce variance and increase statistical power. This approach is advantageous in heterogeneous samples such as hepatocellular carcinoma (HCC), where direct cell-to-cell comparisons may be confounded by compositional shifts and batch effects.

#### 4.3.1. Pseudo-Bulk Aggregation Strategy

To conduct DGE, we aggregated raw count matrices across biological replicates for each annotated cell type within each condition (disease-condition cell types vs. healthy-condition cell types). Aggregation was performed on a per-sample basis, yielding one pseudo-bulk profile per cell type per individual. This approach preserves intra-individual variability and avoids overfitting to donor-specific transcriptional patterns. Library sizes were normalized using the trimmed mean of M-values (TMM) method, and genes with low expression (counts-per-million <1 in >75% of pseudo-bulk samples) were excluded to minimize noise.

Pairwise differential expression was then computed using quasi-likelihood F-tests, controlling for false discovery rate (FDR) using the Benjamini–Hochberg method. Genes with an adjusted *p*-value < 0.05 and an absolute $\log_{2}$ fold change > 1 were considered to be significantly differentially expressed. The resulting gene lists were visualized with bar plots and heatmaps and subsequently used for functional enrichment analyses (Figure S1 and Figure 4).

#### 4.3.2. Pathway and Functional Enrichment Analysis

To contextualize transcriptional differences in terms of biological function, we conducted Gene Set Enrichment Analysis (GSEA) [83] and Overrepresentation Analysis (ORA) using GO biological processes and molecular functions. Hallmark gene sets were drawn from the Molecular Signatures Database (MSigDB) [84].

GSEA was performed using the fgsea package, which ranks all expressed genes by their test statistic and evaluates whether gene sets are overrepresented at the extremes of the ranking. ORA, on the other hand, was applied to the subset of statistically significant differentially expressed genes using the clusterProfiler package, with hypergeometric testing and FDR correction.

This dual enrichment strategy provided complementary insights: GSEA captured subtle coordinated shifts in expression (even without individual gene significance), while ORA highlighted sharply deregulated biological programs. Enrichment results were reported as normalized enrichment scores (NES), adjusted p-values (FDR), and leading-edge subsets, which identify the core drivers of each pathway signal.

#### 4.3.3. Validation and Visualization

To validate the robustness of our DGE findings, we performed sensitivity analyses including alternative normalization strategies (DESeq2’s median-of-ratios method), and subsampling to assess stability across different donor subsets. Additionally, UMAP embeddings were overlaid with expression gradients of top DEGs to confirm consistency with cluster-level annotation. Where applicable, we cross-referenced DEGs with known marker genes and pathway databases such as CellMarker and PanglaoDB.

Enrichment results were visualized using dotplots, network diagrams (via EnrichmentMap), and chord diagrams to depict relationships between pathways and cell types. These visual summaries enabled the interpretation of complex transcriptional programs at a systems level, helping to identify candidate therapeutic targets or diagnostic markers associated with malignant transformation or immune dysregulation.

### 4.4. Cancer Stem Cell Identification and Scoring

To identify cancer stem cell (CSC)-like subpopulations within the malignant cell compartment, we constructed a composite gene signature integrating three evidence layers. First, canonical HCC CSC surface markers (*CD44*, *EPCAM*, *PROM1*, *ALDH1A1*, *CD24*) were included based on the established literature [25]. Second, we incorporated the 17-gene CNV-based stemness signature derived by Ding et al. (2025) [25] through pseudotime trajectory analysis of HCC scRNA-seq data (GSE222791). Third, developmental genes associated with CSC trajectory initiation identified by Lin et al. (2024) [24] were added (*HSPB1*, *ADH4*, *FTH1*, *APCS*). The final signature comprised 34 genes detected in our dataset.

Cell-level scoring was performed using the score_genes function in Scanpy v1.9.3 [69], which computes the mean expression of the signature genes minus a background set of randomly sampled control genes matched for expression level. Cells in the top 30th percentile of CSC scores were classified as CSC-high; the remainder were classified as CSC-low. Leiden clustering at a resolution of 0.8 was applied within the malignant cell subset using the scVI latent representation as input to the *k*-nearest-neighbor graph (k=15). Differential expression between CSC-high and CSC-low cells was assessed with the Wilcoxon rank-sum test, and top markers were identified by score and adjusted *p*-value (Benjamini–Hochberg). Metabolic profiling compared the mean expression of OXPHOS, immune effector, Wnt, and Hedgehog gene panels between the two groups.

### 4.5. Consensus-Based Inference of Intercellular Communication

Intercellular communication within the hepatocellular carcinoma (HCC) microenvironment was inferred using LIANA+, adopting an all-vs.-all design to capture the signaling relationships among annotated cell populations. The analysis combined four complementary tools—CellChat [85], CellPhoneDB [86,87], SingleCellSignalR [88], and NATMI [89]—within a unified rank aggregation scheme implemented in LIANA+ v1.7.1 [31]. Each method estimates interaction strength and specificity through different statistical models and gene expression thresholds, allowing a more reliable and biologically grounded evaluation of ligand–receptor activity across the tissue.

The workflow consisted of three main steps: (i) independent inference of ligand–receptor interactions by each method, (ii) assessment of overlap between methods using the Jaccard similarity index, and (iii) aggregation of ranked outputs into a consensus model. The Jaccard index quantified the proportion of shared top-ranked interactions between methods relative to their combined total, with higher values indicating stronger agreement. This step ensured that the consensus relied on reproducible interaction patterns rather than the bias of a single algorithm.

Consensus scores were then derived through a rank aggregation procedure that combined magnitude and specificity across all methods. Magnitude represented the relative interaction strength inferred from ligand and receptor expression levels, while specificity captured the restriction of each signal to particular sender–receiver pairs. The final score prioritized interactions that were strong, specific, and consistently identified across methods, looking for a robust representation of communication in the dataset.

To minimize false positives, an expression filter was applied, retaining only ligand–receptor pairs where at least 10% of cells in both the sender and receiver populations expressed the corresponding subunits. For complexes with multiple subunits, the lowest expression proportion across all components was used to exclude partial or incomplete interactions. This is relevant in liver tissue, where incomplete receptor expression may lead to artefactual predictions.

After aggregation, interaction matrices were generated to quantify the number and strength of connections between sender–receiver pairs. These matrices summarized dominant communication routes and recurrent signaling relationships within the tumor microenvironment.

#### Intercellular Communication Among Key Hepatic Populations

To refine the consensus analysis and highlight functionally relevant signaling events, we performed a focused evaluation restricted to the main hepatic and tumor-associated populations: hepatocytes, malignant hepatocytes, tumor-associated macrophages (TAMs), and tumor endothelial cells (TECs). These groups were selected for their central roles in tumor metabolism, immune modulation, and vascular remodeling. The analysis used the same consensus framework described above, but it limited the comparison space to interactions involving these populations as either signal senders or receivers.

The dataset was divided into defined sender–receiver groups in which hepatocytes, malignant cells, TAMs, and TECs acted as primary signal sources, while all other annotated populations were retained as potential targets. This approach allowed us to examine how these key cell types communicate within and beyond their own compartments, to clarify their contribution to the tumor microenvironment.

Each subset was analyzed under the same multi-method consensus procedure integrating CellChat, CellPhoneDB, SingleCellSignalR, and NATMI. We applied expression filters to retain only interactions with at least 10% of cells expressing both ligand and receptor components, preventing the inclusion of incomplete or artefactual signals. Magnitude and specificity scores from each method were then aggregated into a consensus rank, prioritizing interactions that were strong, distinct, and reproducibly detected across tools.

### 4.6. Cross-Validation of Healthy and Diseased Datasets

To assess the robustness and generalization of our automated annotation framework, we implemented cross-validation strategies for both healthy and diseased liver datasets. Given the differences in data availability between conditions, the design of the validation scheme was adapted accordingly, while maintaining a common analytical backbone to ensure comparability.

For healthy liver donors, only a single reference dataset (Tabula Sapiens, Healthy Liver Dataset) was available. We applied a stratified K-fold cross-validation. The dataset was randomly partitioned into five folds while preserving the proportional representation of all annotated cell types. In each iteration, four folds (80% of the cells) were used for model training, and the remaining fold (20%) served as the test set. This stratification guaranteed that both common and rare cell populations were represented across folds and allowed us to evaluate the classifier’s ability to recover the full spectrum of liver-resident cell types under resampling.

For hepatocellular carcinoma (HCC) cohorts, which included multiple patient-derived samples, we implemented a stratified group K-fold cross-validation design. Here, folds were defined at the patient sample level, ensuring that cells from a given donor were never simultaneously present in both the training and testing sets. This design prevented information leakage and enabled evaluation under conditions of inter-patient variability. Stratification by cell type was combined with grouping by patient, resulting in balanced folds that respected both cellular composition and biological independence. To avoid instability in classification metrics, cell types with very low counts or represented in fewer than three patient samples were excluded from fold-level testing but retained in the training pool.

In both healthy and diseased settings, model training used scVI and its semi-supervised extension scANVI, implemented in the scvi-tools framework. Within each training fold, 2000 highly variable genes were selected using the Seurat v3 method [80], with batch-aware correction applied. The scVI model was trained for 200 epochs to learn a latent representation of the data, after which scANVI was initialized with these weights and optimized for an additional 20 epochs. The “Unknown” category was designated as the unlabeled class, allowing the model to accommodate cell types present in the test set but absent from training. Predictions on the held-out fold consisted of discrete labels and soft probability distributions across candidate classes. Posterior probabilities were summarized as confidence scores, and cells with maximum probability below 0.5 were flagged as uncertain predictions.

Additionally, beyond fold-level evaluation, we implemented a consensus framework to integrate results across folds within each condition. Standard deviations across folds were reported to capture variability, and consensus recommendations were generated to flag classes with unstable performance or systematic misclassification. This consensus analysis allowed us to overview the model behavior and identify consistently well-annotated cell types and those requiring further scrutiny.

All of the results, including trained models, prediction tables, and fold-level metrics, were stored in standardized formats (.h5ad, .csv, and .png files can be found in the cross_validation_analysis folder in the Supplementary Materials) to ensure reproducibility and allow for external reanalysis. This approach provided evidence that our annotation strategy is not only internally consistent within a single dataset but also robust to inter-individual variability.

## 5. Conclusions

The convergence of immunometabolic transcriptional reprogramming, coordinated functional polarization of immune and stromal compartments, and qualitative rewiring of intercellular communication circuits reported here collectively supports a model in which HCC is sustained by cooperative cellular ecology rather than by cell-autonomous oncogenic programs. This framing has a practical consequence for the field: effective intervention in HCC may require disrupting network-level dependencies—the reciprocal reinforcement among malignant hepatocytes, TAMs, and TECs—rather than targeting isolated molecular nodes within a single compartment. The cooperative immunometabolic modules identified here, along with the quantitative intercellular network indices that could be derived from them, provide a concrete basis for mechanism-based patient stratification and the rational design of combinatorial therapies, once validated in larger, spatially resolved, and multi-omics cohorts.

## Data Availability

Raw data for this study can be found in the following public access repositories: https://www.ncbi.nlm.nih.gov/geo/query/acc.cgi?acc=GSE242889, https://www.ncbi.nlm.nih.gov/geo/query/acc.cgi?acc=GSE228195, https://cellxgene.cziscience.com/collections/0c8a364b-97b5-4cc8-a593-23c38c6f0ac5, https://www.ncbi.nlm.nih.gov/geo/query/acc.cgi?acc=GSE151530, https://www.ncbi.nlm.nih.gov/geo/query/acc.cgi?acc=GSE189903. All processed data and analysis code are deposited at https://github.com/MiguelDiaz02/scRNAseq_a_pipeline_for_HCC; portal source code is available at https://github.com/MiguelDiaz02/HCC-atlas-portal. An interactive web portal for exploring the integrated atlas (93,032 cells; 19,916 genes) is publicly accessible at https://hcc-atlas-portal.onrender.com, enabling UMAP visualization, gene expression queries, and cell-type-resolved marker browsing without requiring local software installation. All accessed on 24 May 2026.

## Supplementary Materials

The following supporting information can be downloaded at https://www.mdpi.com/article/10.3390/ijms27125397/s1. References [75,90] are cited in the Supplementary Materials.
